# Informed Consent in Shoulder and Elbow Surgery

**DOI:** 10.7759/cureus.92584

**Published:** 2025-09-17

**Authors:** Puru Sadh, Antonio Almeda-Lopez, Benjamin Hershfeld, Dan Monessa, Brandon Klein, Randy M Cohn, Adam D Bitterman

**Affiliations:** 1 Department of Orthopaedic Surgery, Donald and Barbara Zucker School of Medicine at Hofstra/Northwell, Hempstead, USA; 2 Department of Orthopaedic Surgery, Philadelphia College of Osteopathic Medicine, Philadelphia, USA; 3 Department of Orthopaedic Surgery, Northwell Health, New Hyde Park, USA; 4 Department of Orthopaedic Surgery, Northwell Health Huntington Hospital, Huntington, USA; 5 Department of Orthopaedic Surgery, University of Pennsylvania Perelman School of Medicine, Philadelphia, USA; 6 Department of Orthopaedic Surgery, Long Island Jewish Valley Stream, Valley Stream, USA

**Keywords:** elbow surgery, informed consent, malpractice, medicolegal, patient autonomy, shoulder surgery, upper extremity

## Abstract

Informed consent is essential for patient autonomy and shared decision-making but remains inconsistent in orthopaedic surgery. Shoulder and elbow procedures present unique challenges, with complex functional outcomes, diverse patient demographics, and high medicolegal exposure. Despite the clinical and legal importance of informed consent, evidence describing current practices in this subspecialty is limited. This review aimed to synthesize the literature on consent in shoulder and elbow surgery, identify barriers to effective communication, and highlight strategies to improve patient understanding and engagement. Peer-reviewed literature was identified through searches of PubMed, Web of Science, and Scopus from database inception through September 11, 2025. Eligible studies addressed informed consent in shoulder and elbow surgery, including consent quality, patient comprehension, expectations, communication interventions, or medicolegal outcomes. Studies not directly addressing informed consent in this context were excluded. Findings reveal limited quantitative data on consent quality, despite high malpractice rates and frequent citation of inadequate consent in litigation. Key barriers include low health literacy, language discordance, insufficient physician training, and time constraints. Patient expectations vary by occupation, gender, and age, emphasizing the need for individualized consent discussions. Proposed solutions emphasize multifaceted, patient-centered approaches: the teach-back method to confirm comprehension, plain-language explanations supported by visual aids, structured resident training to improve communication, timely access to professional interpreter services, and greater workforce diversity to strengthen rapport. Future research should focus on validating specialty-specific consent frameworks, integrating digital adjuncts such as videos or surgery-specific forms, and adopting quantitative outcome measures to evaluate effectiveness. System-wide implementation of these strategies could enhance autonomy, build trust, reduce medicolegal risk, and improve outcomes in shoulder and elbow surgery.

## Introduction and background

Informed consent (IC) is the dynamic process in which a medical provider educates a patient about the risks, benefits, and reasonable alternatives of a procedure [1]. The IC process begins with the initial consultation and ends with the signing of the consent document prior to the procedure [2]. IC encompasses all discussions during the patient's care, allowing for a patient's thorough understanding and voluntary approval of the proposed procedure. Adequate IC is crucial for preserving patient autonomy and must include discussions regarding five key elements: (1) nature of the procedure, (2) risks and benefits of the procedure, (3) reasonable alternatives, including no intervention, (4) risks and benefits of the alternatives, and (5) assessment of the patient's understanding of the previously stated elements [3]. Therefore, it is imperative that medical providers document the progression of discussions, as IC is not limited to a single encounter [4].

Performing medical intervention without obtaining adequate IC infringes on patient autonomy and may be considered "battery" or "physical assault" [4]. The presence of well-documented IC in the surgeon's office notes correlated with a notable reduction in indemnity risk and was associated with a considerable decrease in the risk of malpractice payment. Conversely, acquiring IC in the hospital ward or preoperative holding area was linked to an elevated indemnity risk [4]. These findings highlight the importance of adequate IC in orthopaedics and underscore the need for continued discussions regarding IC in the preoperative process.

There are unique considerations of IC in shoulder and elbow (SE) surgery that justify special attention. Unlike hip and knee procedures, which primarily restore ambulation and weight-bearing function, SE operations directly affect overhead activity, reach, grip, and fine motor coordination. These functions are critical to employment and activities of daily living [5]. Return-to-work rates after SE surgery are significantly lower in patients with moderate to heavy physical demands compared to those with sedentary or light work, which underscores the occupation-specific impact of functional outcomes [6]. Sustained or frequent overhead work is also a well-established risk factor for shoulder disorders [5]. In addition, inadequate recall of surgical risks and expectations is common among SE patients, even when surgery-specific consent forms or educational videos are used, which makes the consent process more complex [7].

Finally, while orthopaedic surgery overall is among the most litigated specialties, SE procedures face a disproportionate medicolegal burden. This is partly because functional outcomes are highly individualized and difficult to guarantee, which increases the likelihood of unmet expectations. Patients often expect restoration of full strength and dexterity, yet even small postoperative deficits can impair work capacity and quality of life. These mismatches between expectations and achievable results frequently underpin malpractice claims. As a result, lack of IC is cited in approximately 13% of orthopaedic malpractice cases overall and up to 19% of elbow-surgery claims [8,9].

Due to the paucity of literature surrounding the IC process in the orthopaedic subspecialty of SE surgery, this study seeks to answer the following questions: (1) What is the current state of the IC process in SE surgery? (2) What unique factors and expectations must be considered with the treatment of the patient population of SE surgeons? (3) What proposed solutions are there to improve the adequacy of IC?

## Review

Materials and methods

Study Design

This study adopts a narrative review approach to synthesize current evidence on IC in orthopaedic SE surgery. The analysis integrates quantitative data on complication rates, litigation outcomes, and communication interventions with qualitative insights from patient expectations, health literacy, and physician-patient communication literature.

Data Sources and Search Strategy

Peer-reviewed literature was identified through searches of PubMed, Web of Science, and Scopus from database inception through September 11, 2025. The search combined Medical Subject Headings (MeSH) and free-text terms, including "informed consent", "shoulder surgery", "elbow surgery", "upper extremity", "orthopaedic surgery", "malpractice", and "patient autonomy". Reference lists of relevant studies were also manually screened to identify additional eligible articles.

Inclusion and Exclusion Criteria

Studies were included if they met the following criteria: (1) English-language publications focused on IC in SE surgery; (2) addressed at least one of the following: consent quality, patient comprehension, patient expectations, communication interventions, or medicolegal outcomes. Exclusion criteria were (1) studies not directly addressing IC; (2) editorials, commentaries, or conference abstracts without original or review data; and (3) studies focusing exclusively on non-orthopaedic specialties.

Search Results and Synthesis

**Figure 1:**
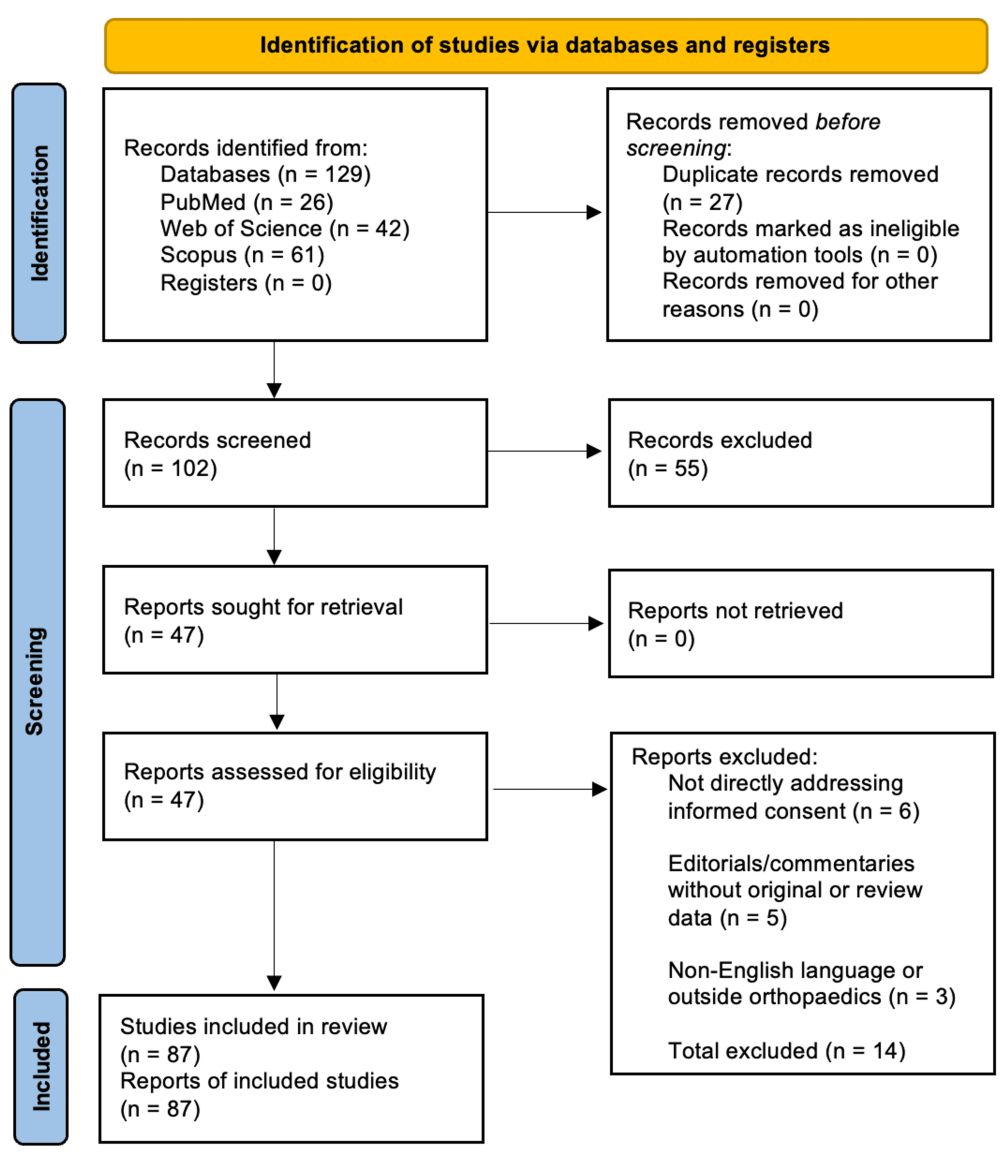
PRISMA flow diagram PRISMA = Preferred Reporting Items for Systematic Reviews and Meta-Analyses.

The initial search identified 129 records (PubMed = 26, Web of Science = 42, Scopus = 61). After removing duplicates, 102 records remained. Following title and abstract screening, 55 records were excluded, leaving 47 full-text articles for assessment. Of these, 33 met the inclusion criteria. An additional 54 articles were identified through manual reference list searching, resulting in 87 total studies included in this narrative synthesis. The selection process is detailed in Figure 1, following the PRISMA (Preferred Reporting Items for Systematic Reviews and Meta-Analyses) flow diagram guidelines.

Given the heterogeneity of study designs and outcomes, a qualitative thematic synthesis was performed. Articles were organized into four domains: current state of IC in SE surgery, unique considerations in patient populations, barriers to effective IC, and proposed solutions to strengthen IC practices.

Quality Assessment

Because the majority of included studies were descriptive or retrospective in nature, no formal risk-of-bias tool was applied. Methodological limitations, including small sample sizes, recall bias, and limited generalizability, are noted where relevant.

Unique considerations in SE surgery

Demographics

SE surgery serves a diverse patient demographic, which underscores the importance of using a shared decision-making approach. Studies have shown that occupation, gender, and age are critical factors influencing outcomes in patients treated by orthopaedic SE surgeons [5,6,10-12].

Occupation has a strong influence on the prevalence of SE disorders. Manual labor is a major risk factor for shoulder injury, and nearly all civilian jobs (99.9%) require the gross manipulation of the hands. Almost half of the United States labor force (45%) works in jobs requiring medium to high physical strength, which involve lifting, standing, and walking [11]. Workers in physically demanding roles such as nursing, caregiving, and teaching undergo surgical interventions at higher rates [12]. Exposure to repetitive mechanical factors, such as lifting heavy objects or frequent overhead activity, is strongly associated with shoulder injuries. In fact, exposure to three or more such factors increases the risk of clinically diagnosed shoulder disorders fourfold [5].

Gender also influences the development of shoulder disorders. Women in physically demanding jobs often face different risks due to biomechanical differences, which require them to exert more effort to complete tasks compared to men [10]. This heightened exertion predisposes women to a greater incidence of SE injuries, particularly in repetitive or strenuous roles [10,12]. Statistically, women account for 29% of elbow arthroscopic procedures, 42% of rotator cuff repairs, and 64% of total shoulder arthroplasty cases [13-15].

Age further affects perioperative and postoperative outcomes. Elderly women have the highest risk of complications after shoulder arthroplasty, while younger men face a fourfold greater lifetime risk of revision surgery [6]. In rotator cuff repair, women often have longer hospital stays and more minor adverse events, despite shorter operative times [16]. Recognizing these demographic differences helps surgeons deliver evidence-based, patient-centered IC discussions.

In addition, the demand for primary shoulder arthroplasty has been increasing across all age groups. Projections estimate a 333.3% rise in younger patients and a 755.4% rise in patients over 55 years old between 2011 and 2030. This increase reflects demographic aging, broader surgical indications, and a greater willingness to pursue arthroplasty with advances in implant design and outcomes [17]. The expected rise in SE procedures further highlights the importance of optimizing the IC process for this population.

Patient Expectations

Clear patient expectations are strongly correlated with improved outcomes across medical specialties, and this is particularly relevant for SE surgery [18,19]. Studies show that patients undergoing these procedures commonly expect pain relief, prevention of disability, better sleep comfort, and improved range of motion [20-22]. When these expectations are met, satisfaction with surgical outcomes increases significantly [18].

Compared to other orthopaedic procedures, SE surgeries often involve additional considerations. A study comparing shoulder surgery with other orthopaedic operations found that patients' general health and employment status had a substantial effect on postoperative satisfaction [23]. In other words, many SE patients view outcomes not only in terms of functional improvement but also in how their conditions affect overall health and ability to work.

Expectations differ by demographic group. For example, male patients often prioritize sports participation, uninterrupted sleep, and job retention before undergoing shoulder arthroplasty [24]. In contrast, female patients frequently place greater emphasis on household chores and activities of daily living [24,25]. Younger patients tend to have higher expectations for postoperative improvement than older patients [20]. These differences highlight the importance of tailoring IC discussions to each patient's goals and values.

Incorporating individualized, evidence-based information into the IC process can enhance patient comprehension and satisfaction with surgical outcomes [26]. These personalized discussions should be integrated into broader strategies, such as using plain language, visual aids, and structured communication tools, to further improve IC quality. The interplay between patient demographics, expectations, and the evolving nature of SE surgery emphasizes the critical role of patient-centered consent in optimizing outcomes.

Current state of IC in SE surgery

Anatomic total shoulder arthroplasty (aTSA), reverse shoulder arthroplasty (RSA), shoulder arthroscopy (SA), and ulnar nerve transposition (UNT) are among the most frequently performed procedures in SE surgery [6,27]. Despite their rise in popularity, complications still occur (Table 1). Reported rates in the literature range from 7.1 to 11.5% for aTSA/RSA, 1.0 to 7.9% for SA, and 3 to 9.6% for UNT [3,28-30]. In SE surgery, these complication risks may strongly influence a patient's decision to proceed with an operation. Therefore, it is essential that the IC process includes disclosure of achievable outcomes, the potential impact of complications on recovery, and available alternatives that align with patient goals.

**Table 1:** Reported postoperative complications and incidence rates following common shoulder and elbow procedures Data adapted from Bohsali et al. (2017) (shoulder arthroplasty, n = 7,484) [31], Shin et al. (2018) (shoulder arthroscopy, n = 27,072) [3], and Zhang et al. (2016) (ulnar nerve transposition, n = 115) [30]. Complication rates represent reported incidence in respective cohorts. TSA = total shoulder arthroplasty; RSA = reverse shoulder arthroplasty; VTE = venous thromboembolism; MACN = medial antebrachial cutaneous nerve; SA = shoulder arthroscopy; UNT = ulnar nerve transposition.

Complication	No. of Shoulders (TSA/RSA)	Rate % (TSA/RSA)	Rate % (SA)	Rate % (UNT)
Instability	243	3.2	-	-
Periprosthetic Fracture	161	2.2	-	-
Infection	135	1.8	-	-
Component Loosening	210	2.8	-	-
Glenoid Wear	78	1	-	-
Neural Injury	71	0.95	-	-
Acromial/Scapular Spine Fracture	40	0.53	-	-
Hematoma	24	0.32	-	-
Deltoid Injury	7	0.09	-	-
Rotator Cuff Repair	4	0.05	-	-
VTE Events	5	0.07	-	-
Total Complications	-	11	-	-
Stiffness/Arthrofibrosis	-	-	2.22	-
Persistent Pain	-	-	1.86	-
Infection	-	-	0.65	-
Nerve Palsy	-	-	0.51	-
Respiratory Failure	-	-	0.3	-
VTE	-	-	0.3	-
Pneumonia	-	-	0.11	-
Cerebrovascular Accident	-	-	0.04	-
Death	-	-	0.08	-
Overall (Select Complications)	-	-	7.9	-
Persistent Cubital Tunnel Syndrome	-	-	-	7.8
Postoperative Infection	-	-	-	0.9
MACN Injury	-	-	-	0.9
Overall	-	-	-	9.6

Patient autonomy has important implications in orthopaedic surgery, which remains one of the most frequently litigated medical specialties. Approximately 15% of practicing orthopaedic surgeons face litigation claims each year [32,33]. Because objective measures of IC quality in upper extremity surgery are limited, malpractice data can highlight areas for improvement. Of the malpractice claims filed against shoulder surgeons, 13% were specifically related to inadequate IC [8]. This demonstrates that even though most hospitals require a formal IC process, inadequate consent remains a common allegation in litigation.

The literature consistently emphasizes the need to strengthen IC practices across invasive medical specialties. In a study of patients undergoing SA, Gibson et al. found that recall of surgical risks was limited, with patients remembering an average of only 2.55 risks after reviewing a standard consent form and 3.45 after reviewing a surgery-specific form. Although the surgery-specific form improved recall, long-term retention remained poor [7]. Furthermore, patients must understand how comorbidities and prior interventions can alter their personal risk profile [34]. For example, a recent systematic review found that preoperative corticosteroid injections, particularly when administered repeatedly or within three months of surgery, were consistently associated with an increased risk of postoperative infection following both SA and arthroplasty [35]. These patient-specific factors illustrate how routine clinical decisions can substantially change complication risk and why they must be clearly communicated during the IC process. Taken together, the combination of litigation risk and the unique complexities of UE surgery highlights the urgent need to optimize the IC process in SE procedures [36-38].

Barriers to obtaining adequate IC

The IC process faces many challenges, including a lack of physician training, insufficient guidelines, low health literacy, limited patient education, racial and cultural differences, and language discordance [39-44]. These issues undermine patients' ability to achieve a fundamental understanding of proposed procedures [45].

Inadequate Training

A survey of 2,523 first-year residents by Raymond et al. found that 83% were responsible for obtaining IC for minor procedures. Yet, only 26% did so under the supervision of an attending physician. Similarly, 41% obtained IC for major procedures, with just 37% reporting that an attending was present [40,46,47]. These findings highlight the limited oversight and formal instruction early in training.

Consistent with this, a survey of 150 physicians reported that 44.7% rated their ethics education as "fair" or "poor," with most instruction occurring through informal conversations [48]. Likewise, only 33.5% of orthopaedic residents reported receiving formal IC training, and just 4.2% disclosed all essential information during the process [49]. Collectively, these data emphasize that inadequate training contributes to deficient consent practices, exposing surgeons to adverse patient outcomes and potential litigation.

Low Health Literacy

Low health literacy is common among racially and ethnically diverse groups, older adults, and individuals with poor self-rated health, placing them at higher risk for poor outcomes [50,51]. IC documents are recommended at a sixth-eighth grade level, but most remain more complex, emphasizing documentation rather than comprehension [52-54]. Without reform, this barrier perpetuates disparities and avoidable costs [55].

Foreign Language Barriers

IC should occur in a patient's primary language, yet communication is often hindered by complex medical terminology and cultural disconnects [56]. Interpreters may reframe dialogue to match cultural norms without notifying the clinician [45,57,58]. Although federal regulations require language assistance, enforcement is weak. Physicians' use of professional interpreters depends heavily on availability within 15 minutes [59-62]. In their absence, ad hoc interpreters (family members, bilingual staff, or physicians' limited language skills) are often used, risking omission of critical information [62-64]. Such breakdowns in communication not only compromise autonomy but have also been cited as contributing factors in malpractice claims involving inadequate consent [58].

Interpersonal and Sociocultural Factors

Non-verbal cues and bedside manner influence patient comprehension and trust [65,66]. Patients with SE conditions often report higher anxiety than other orthopaedic populations, and empathy and trustworthiness are critical to improving patient understanding and compliance [34,67,68]. Cuevas and O'Brien found that among patients identifying as African American, stronger racial identity was associated with greater mistrust of the healthcare system [69]. Similarly, resident race and ethnicity have been shown to influence patient preferences in clinical interactions [70]. Overall, sociopolitical beliefs significantly affect autonomy in ways that are often underrecognized [58].

Time Constraints

Orthopaedic IC discussions average 16.1 minutes, ranging from 3 to 76 minutes [42]. Longer discussions are associated with greater trust and comprehension, but no universally recommended duration has been established [71]. The optimal length is likely procedure-specific, depending on patient questions, preoperative severity of function, comorbidities, and complexity of the proposed surgery. Importantly, rushed or overly brief encounters can leave patients with a partial or inaccurate understanding, undermining the validity of IC and increasing medicolegal vulnerability [42,71]. Ensuring adequate time for patient dialogue remains an essential but underexplored component of IC quality.

Proposed solutions to barriers

Improving the IC process requires a multifaceted strategy to address the barriers described above. Approaches include the teach-back method to assess patient comprehension, structured training for residents and surgeons, tailored strategies for low health literacy populations, communication tools to support patient engagement, and methods to overcome language barriers [7,40,71-74].

Teach-Back and Relationship-Centered Communication

The teach-back method creates a patient-centered environment by having patients restate their understanding, allowing clinicians to identify any misunderstandings and reinforce key points [71]. Yu and Pun outline strategies such as assessing comprehension, clarifying information, addressing psychological concerns, and repeating crucial details [74]. This approach improves recall and trust, enhancing adherence [7,71]. Relationship-centered care emphasizes empathy and mutuality; core principles include shared affect, genuine connection, and positive physician demeanor [73,75]. Reviewing postoperative expectations both before and after surgery further supports adherence [72].

Improved Training

Structured training programs can improve IC delivery. Koller et al. recommend targeted lectures followed by standardized patient encounters with faculty feedback, which improved residents' confidence and skill [40]. Yet, surveys show that many residents still lack consistent instruction, highlighting the need for broader integration of formal IC training into curricula [48,49]. Without such preparation, surgeons may omit critical disclosures, predisposing them to patient dissatisfaction and litigation [49]. Beyond educational benefits, structured training may also reduce downstream medicolegal risk by standardizing disclosure of risks, benefits, and alternatives across providers [48,49].

Strategies for Low Health Literacy Populations

Effective strategies include plain language, focusing on critical messages, and incorporating visual aids [48]. Patients better understand when discussions are individualized and led by the primary surgeon [48,76]. Educational materials should match a sixth-eighth grade reading level [52,77]. However, many materials exceed this level, as shown in various orthopaedic societies' patient documents [78]. Visual aids, such as illustrations and bold formatting, improve comprehension and confidence [41,79]. The IC process should prioritize patient education rather than mere documentation [41].

Diversity in the Workforce

Many patients with low health literacy are non-White individuals, and literature suggests that patients with a similar appearance or race as the physician report greater healthcare satisfaction [77,80]. A cross-sectional analysis of the Press Ganey survey, used by many practices to evaluate patient experience, demonstrated higher satisfaction when patients were treated by physicians of a concordant racial or ethnic background [23]. These findings emphasize the importance of increasing diversity within orthopaedic surgery, including subspecialties such as SE surgery, where patient populations are demographically diverse and outcomes are strongly influenced by functional expectations [13,20].

Interpersonal Relationships

Bedside manner directly affects comprehension, trust, and adherence [81]. Rapport between patient and surgeon increases the likelihood of agreement and satisfaction [71,74,82]. Physicians who demonstrate empathy, attentiveness, and follow-up improve satisfaction and compliance [81]. Preoperative discussions of rehabilitation and postoperative plans increase adherence, while poor relationships are associated with litigation [72,83]. Because many SE patients are manual workers requiring high dexterity, surgeons must emphasize functional risks during IC. However, assessing preoperative functional status is difficult [5,12,84]. Techniques such as teach-back, plain language, and visual aids should be applied specifically to upper extremity function and rehabilitation [85].

Interpreter Services

Where language discordance exists, bilingual clinicians are preferred. When unavailable, professional interpreters remain critical [45,86]. Hospitals that implemented bedside interpreter phones reported better patient-reported IC experiences, while delays in access reduced utilization [62,87]. In contrast, reliance on ad hoc interpreters such as family members, bilingual staff, or physicians' limited skills often results in miscommunication of crucial details [62,63]. Cultural concordance further enhances trust, emphasizing the value of interpreters who understand patients' sociopolitical and health contexts [58]. Conversely, failure to use professional interpreters has been associated with poorer comprehension, greater dissatisfaction, and increased medicolegal exposure [62,63].

Future directions

Emerging digital and visual adjuncts, including surgery-specific consent forms, video-based education, and stereoscopic visualization, have shown promise in improving comprehension and retention of surgical risks, although these approaches remain underexplored in SE surgery [7,79]. Comparisons with other orthopaedic subspecialties, particularly hip and knee arthroplasty, demonstrate that differences in functional outcomes influence patient expectations and the consent process. Lower extremity procedures emphasize ambulation and weight-bearing, whereas SE surgery more directly affects overhead reach, dexterity, and fine motor control [72]. International perspectives further reveal variability in consent standards, cultural values, and legal frameworks, underscoring that best practices may differ across healthcare systems [57,58]. Taken together, these gaps highlight the need for SE-specific consent frameworks that integrate digital adjuncts, draw insights from other subspecialties, and are adaptable across diverse clinical and cultural settings.

## Conclusions

IC for SE surgery remains inconsistently executed, with substantial variability in patient comprehension, communication practices, and medicolegal outcomes. This review highlights a clear gap in high-quality evidence, as most studies are descriptive and few employ validated tools to assess IC quality or patient understanding. Recurrent barriers include low health literacy, language discordance, inadequate training, and limited discussion time, all of which undermine the consent process. Promising strategies such as the teach-back method, plain language, visual aids, structured resident education, and timely interpreter services may help strengthen patient comprehension and engagement.

Looking forward, future work should focus on validating specialty-specific consent frameworks, integrating digital adjuncts, and adopting quantitative outcome measures to evaluate effectiveness. Identifying standardized, patient-centered approaches will be critical for enhancing autonomy, improving trust, and reducing medicolegal risk in SE surgery.

## References

[1] Hershfeld B, Klein B, White PB, Mont MA, Bitterman AD (2024). Informed consent in orthopaedic surgery: a primer. J Bone Joint Surg Am.

[2] Kadam RA (2017). Informed consent process: a step further towards making it meaningful!. Perspect Clin Res.

[3] Shin JJ, Popchak AJ, Musahl V, Irrgang JJ, Lin A (2018). Complications after arthroscopic shoulder surgery: a review of the American Board of Orthopaedic Surgery database. J Am Acad Orthop Surg Glob Res Rev.

[4] Satyanarayana Rao KH (2008). Informed consent: an ethical obligation or legal compulsion?. J Cutan Aesthet Surg.

[5] Linaker CH, Walker-Bone K (2015). Shoulder disorders and occupation. Best Pract Res Clin Rheumatol.

[6] Craig RS, Lane JC, Carr AJ, Furniss D, Collins GS, Rees JL (2019). Serious adverse events and lifetime risk of reoperation after elective shoulder replacement: population based cohort study using hospital episode statistics for England. BMJ.

[7] Gibson AW, Cahill A, Piggott R, Cashman J, O'Briain D (2023). Patient recall of informed consent at 4 weeks following arthroscopic shoulder surgery with standardised versus the surgery-specific consent. J Shoulder Elbow Surg.

[8] Lynch JC, Radack TM, Stenson JF, Riebesell SA, Austin LS (2022). Malpractice against shoulder surgeons: what the data say. J Shoulder Elbow Surg.

[9] Brito E, Sherman N, Mahoney AP (2024). A 23-year analysis of litigation in orthopedic elbow surgery. J Shoulder Elbow Surg.

[10] Barth KA, Eliasberg CD, Sutton KM (2022). Sex-specific considerations for shoulder instability and adhesive capsulitis in females. J Orthop Orthop Surg.

[11] Biswas A, Harbin S, Irvin E (2022). Differences between men and women in their risk of work injury and disability: a systematic review. Am J Ind Med.

[12] Linde F, Rydberg M, Zimmerman M (2022). Surgically treated carpal tunnel syndrome and ulnar nerve entrapment at the elbow in different occupations and their effect on surgical outcome. J Occup Environ Med.

[13] Linker JA, Eberlin CT, Naessig SA (2023). Racial disparities in arthroscopic rotator cuff repair: an analysis of utilization and perioperative outcomes. JSES Int.

[14] Leong NL, Cohen JR, Lord E, Wang JC, McAllister DR, Petrigliano FA (2015). Demographic trends and complication rates in arthroscopic elbow surgery. Arthroscopy.

[15] Westermann RW, Pugely AJ, Martin CT, Gao Y, Wolf BR, Hettrich CM (2015). Reverse shoulder arthroplasty in the United States: a comparison of national volume, patient demographics, complications, and surgical indications. Iowa Orthop J.

[16] Rudisill SS, Eberlin CT, Kucharik MP, Linker JA, Naessig SA, Best MJ, Martin SD (2022). Sex differences in utilization and perioperative outcomes of arthroscopic rotator cuff repair. JSES Int.

[17] Padegimas EM, Maltenfort M, Lazarus MD, Ramsey ML, Williams GR, Namdari S (2015). Future patient demand for shoulder arthroplasty by younger patients: national projections. Clin Orthop Relat Res.

[18] Henn RF 3rd, Kang L, Tashjian RZ, Green A (2007). Patients' preoperative expectations predict the outcome of rotator cuff repair. J Bone Joint Surg Am.

[19] Swarup I, Henn CM, Gulotta LV, Henn RF 3rd (2019). Patient expectations and satisfaction in orthopaedic surgery: a review of the literature. J Clin Orthop Trauma.

[20] Henn RF 3rd, Ghomrawi H, Rutledge JR, Mazumdar M, Mancuso CA, Marx RG (2011). Preoperative patient expectations of total shoulder arthroplasty. J Bone Joint Surg Am.

[21] Oh JH, Yoon JP, Kim JY, Kim SH (2012). Effect of expectations and concerns in rotator cuff disorders and correlations with preoperative patient characteristics. J Shoulder Elbow Surg.

[22] Rauck RC, Swarup I, Chang B, Dines DM, Warren RF, Gulotta LV, Henn RF 3rd (2018). Effect of preoperative patient expectations on outcomes after reverse total shoulder arthroplasty. J Shoulder Elbow Surg.

[23] Takeshita J, Wang S, Loren AW, Mitra N, Shults J, Shin DB, Sawinski DL (2020). Association of racial/ethnic and gender concordance between patients and physicians with patient experience ratings. JAMA Netw Open.

[24] Hung NJ, Wong SE (2022). Gender influences on shoulder arthroplasty. Curr Rev Musculoskelet Med.

[25] Jawa A, Dasti U, Brown A, Grannatt K, Miller S (2016). Gender differences in expectations and outcomes for total shoulder arthroplasty: a prospective cohort study. J Shoulder Elbow Surg.

[26] Howard N, Cowan C, Ahluwalia R, Wright A, Hennessy M, Jackson G, Platt S (2018). Improving the consent process in foot and ankle surgery with the use of personalized patient literature. J Foot Ankle Surg.

[27] Kinaci A, Neuhaus V, Ring D (2015). Surgical procedures of the elbow: a nationwide cross-sectional observational study in the United States. Arch Bone Jt Surg.

[28] Huddleston HP, Mehta N, Polce EM, Williams BT, Fu MC, Yanke AB, Verma NN (2021). Complication rates and outcomes after outpatient shoulder arthroplasty: a systematic review. JSES Int.

[29] Wade RG, Griffiths TT, Flather R, Burr NE, Teo M, Bourke G (2020). Safety and outcomes of different surgical techniques for cubital tunnel decompression: a systematic review and network meta-analysis. JAMA Netw Open.

[30] Zhang D, Earp BE, Blazar PE (2016). Complication rates of cubital tunnel surgery: in situ cubital tunnel release compared with ulnar nerve transposition: level 3 evidence. J Hand Surg Am.

[31] Bohsali KI, Bois AJ, Wirth MA (2017). Complications of shoulder arthroplasty. J Bone Joint Surg Am.

[32] Rynecki ND, Coban D, Gantz O (2018). Medical malpractice in orthopedic surgery: a Westlaw-based demographic analysis. Orthopedics.

[33] Schaffer AC, Jena AB, Seabury SA, Singh H, Chalasani V, Kachalia A (2017). Rates and characteristics of paid malpractice claims among US physicians by specialty, 1992-2014. JAMA Intern Med.

[34] Zhou WJ, Wan QQ, Liu CY, Feng XL, Shang SM (2017). Determinants of patient loyalty to healthcare providers: an integrative review. Int J Qual Health Care.

[35] Lucenti L, Panvini FM, de Cristo C, Rapisarda D, Sapienza M, Testa G, Pavone V (2024). Do preoperative corticosteroid injections increase the risk of infection after shoulder arthroscopy or shoulder arthroplasty? A systematic review. Healthcare (Basel).

[36] Jildeh TR, Abbas MJ, Hengy MH, O'Brien H, Gani GS, Okoroha KR (2021). Informed consent for the orthopaedic surgeon. JBJS Rev.

[37] Pai SN (2021). Informed consent in orthopaedic cases. Int J Res Orthop.

[38] Porcellini G, Campi F, Paladini P, Rossi P, Lollino N (2008). Informed consent in shoulder surgery. Chir Organi Mov.

[39] Kesselheim JC, Johnson J, Joffe S (2008). Pediatricians' reports of their education in ethics. Arch Pediatr Adolesc Med.

[40] Koller SE, Moore RF, Goldberg MB (2017). An informed consent program enhances surgery resident education. J Surg Educ.

[41] Simonds VW, Garroutte EM, Buchwald D (2017). Health literacy and informed consent materials: designed for documentation, not comprehension of health research. J Health Commun.

[42] Braddock C 3rd, Hudak PL, Feldman JJ, Bereknyei S, Frankel RM, Levinson W (2008). "Surgery is certainly one good option": quality and time-efficiency of informed decision-making in surgery. J Bone Joint Surg Am.

[43] Cawich SO, Barnett AT, Crandon IW, Drew SD, Gordon-Strachan G (2013). From the patient's perspective: is there a need to improve the quality of informed consent for surgery in training hospitals?. Perm J.

[44] O'Sullivan S, Crippen C, Ponich T (2002). Are patients informed when they consent to ERCP?. Can J Gastroenterol.

[45] Simon CM, Zyzanski SJ, Durand E, Jimenez XF, Kodish ED (2006). Interpreter accuracy and informed consent among Spanish-speaking families with cancer. J Health Commun.

[46] Raymond MR, Mee J, King A, Haist SA, Winward ML (2011). What new residents do during their initial months of training. Acad Med.

[47] McClean KL, Card SE (2004). Informed consent skills in internal medicine residency: how are residents taught, and what do they learn?. Acad Med.

[48] Joseph G, Lee R, Pasick RJ, Guerra C, Schillinger D, Rubin S (2019). Effective communication in the era of precision medicine: a pilot intervention with low health literacy patients to improve genetic counseling communication. Eur J Med Genet.

[49] Alomar AZ (2021). Confidence level, challenges, and obstacles faced by orthopedic residents in obtaining informed consent. J Orthop Surg Res.

[50] Bennett IM, Chen J, Soroui JS, White S (2009). The contribution of health literacy to disparities in self-rated health status and preventive health behaviors in older adults. Ann Fam Med.

[51] Schillinger D (2021). Social determinants, health literacy, and disparities: Intersections and controversies. Health Lit Res Pract.

[52] Hadden KB, Prince LY, Moore TD, James LP, Holland JR, Trudeau CR (2017). Improving readability of informed consents for research at an academic medical institution. J Clin Transl Sci.

[53] Grady C (2015). Enduring and emerging challenges of informed consent. N Engl J Med.

[54] Lorenzen B, Melby CE, Earles B (2008). Using principles of health literacy to enhance the informed consent process. AORN J.

[55] Lans A, Bales JR, Fourman MS, Borkhetaria PP, Verlaan JJ, Schwab JH (2023). Health literacy in orthopedic surgery: a systematic review. HSS J.

[56] McCarthy DM, Leone KA, Salzman DH, Vozenilek JA, Cameron KA (2012). Language use in the informed consent discussion for emergency procedures. Teach Learn Med.

[57] Edwards SJ, Lilford RJ, Thornton J, Hewison J (1998). Informed consent for clinical trials: in search of the "best" method. Soc Sci Med.

[58] Kaufert JM, O'Neil JD (1990). Biomedical rituals and informed consent: native Canadians and the negotiation of clinical trust. Social Science Perspectives on Medical Ethics.

[59] Chen AH, Youdelman MK, Brooks J (2007). The legal framework for language access in healthcare settings: title VI and beyond. J Gen Intern Med.

[60] Avra T, Cordova D, Taira B, Torres JR (2024). Utilization and perceptions of language assistance services by medical trainees: a pathway to language certification. AIMS Public Health.

[61] Koh HK, Gracia JN, Alvarez ME (2014). Culturally and linguistically appropriate services—advancing health with CLAS. N Engl J Med.

[62] Patel DN, Wakeam E, Genoff M, Mujawar I, Ashley SW, Diamond LC (2016). Preoperative consent for patients with limited English proficiency. J Surg Res.

[63] Baker DW, Parker RM, Williams MV, Coates WC, Pitkin K (1996). Use and effectiveness of interpreters in an emergency department. JAMA.

[64] Levine C (2006). Use of children as interpreters. JAMA.

[65] Brady RE, Braz AN (2023). Challenging interactions between patients with severe health anxiety and the healthcare system: a qualitative investigation. J Prim Care Community Health.

[66] Legg AM, Andrews SE, Huynh H, Ghane A, Tabuenca A, Sweeny K (2015). Patients' anxiety and hope: predictors and adherence intentions in an acute care context. Health Expect.

[67] Beleckas CM, Wright M, Prather H, Chamberlain A, Guattery J, Calfee RP (2018). Relative prevalence of anxiety and depression in patients with upper extremity conditions. J Hand Surg Am.

[68] Wang Y, Wu Q, Wang Y, Wang P (2022). The effects of physicians' communication and empathy ability on physician-patient relationship from physicians' and patients' perspectives. J Clin Psychol Med Settings.

[69] Cuevas AG, O'Brien K (2019). Racial centrality may be linked to mistrust in healthcare institutions for African Americans. J Health Psychol.

[70] Corwin AM, Rajkumar JN, Markovitz BJ (2019). Association of preoperative disclosure of resident roles with informed consent for cataract surgery in a teaching program. JAMA Ophthalmol.

[71] Seely KD, Higgs JA, Butts L, Roe JM, Merrill CB, Zapata I, Nigh A (2022). The "teach-back" method improves surgical informed consent and shared decision-making: a proof of concept study. Patient Saf Surg.

[72] Bakaa N, Chen LH, Carlesso L, Richardson J, Shanthanna H, Macedo L (2022). Understanding barriers and facilitators of exercise adherence after total-knee arthroplasty. Disabil Rehabil.

[73] Nassar AK, Weimer-Elder B, Yang R (2023). Developing an inpatient relationship centered communication curriculum (I-RCCC) rounding framework for surgical teams. BMC Med Educ.

[74] Yu QJ, Pun J (2023). Promoting patient engagement in medical informed consent - a qualitative study of Chinese doctors' communication strategies. Health Commun.

[75] Beach MC, Inui T (2006). Relationship-centered care. A constructive reframing. J Gen Intern Med.

[76] Steinberg A (2009). Disclosure of information and informed consent: ethical and practical considerations. J Child Neurol.

[77] Cutilli CC, Bennett IM (2009). Understanding the health literacy of America: results of the National Assessment of Adult Literacy. Orthop Nurs.

[78] Ó Doinn T, Broderick JM, Abdelhalim MM, Quinlan JF (2021). Readability of patient educational materials in pediatric orthopaedics. J Bone Joint Surg Am.

[79] Hertzsprung N, Krantchev K, Picht T (2023). Personalized surgical informed consent with stereoscopic visualization in neurosurgery—real benefit for the patient or unnecessary gimmick?. Acta Neurochir (Wien).

[80] Ku L, Vichare A (2023). The association of racial and ethnic concordance in primary care with patient satisfaction and experience of care. J Gen Intern Med.

[81] Zulman DM, Haverfield MC, Shaw JG (2020). Practices to foster physician presence and connection with patients in the clinical encounter. JAMA.

[82] Ahmadi Z, Haghmoradi M, Baradaran A, Rahimi R (2023). What makes patients stick with an orthopedic surgeon?. SurgiColl.

[83] Moore PJ, Adler NE, Robertson PA (2000). Medical malpractice: the effect of doctor-patient relations on medical patient perceptions and malpractice intentions. West J Med.

[84] Weiss AC, Wiedeman G Jr, Quenzer D, Hanington KR, Hastings H 2nd, Strickland JW (1995). Upper extremity function after wrist arthrodesis. J Hand Surg Am.

[85] Grandizio LC, Gehrman MD, Graham J, Dwyer CL, Sharma J, Goldberg S, Klena JC (2021). The ability of upper extremity surgeons to assess patient's functional status. J Hand Surg Am.

[86] Villalobos BT, Bridges AJ, Anastasia EA, Ojeda CA, Rodriguez JH, Gomez D (2016). Effects of language concordance and interpreter use on therapeutic alliance in Spanish-speaking integrated behavioral health care patients. Psychol Serv.

[87] Lee JS, Pérez-Stable EJ, Gregorich SE, Crawford MH, Green A, Livaudais-Toman J, Karliner LS (2017). Increased access to professional interpreters in the hospital improves informed consent for patients with limited English proficiency. J Gen Intern Med.

